# Progressive expression of *PPARGC1α* is associated with hair miniaturization in androgenetic alopecia

**DOI:** 10.1038/s41598-019-43998-7

**Published:** 2019-06-19

**Authors:** Bryan Siu-Yin Ho, Candida Vaz, Srinivas Ramasamy, Elaine Guo Yan Chew, Jameelah Sheik Mohamed, Huma Jaffar, Axel Hillmer, Vivek Tanavde, Mei Bigliardi-Qi, Paul Lorenz Bigliardi

**Affiliations:** 1 Experimental Dermatology Group, Institute of Medical Biology, A*STAR (Agency for Science, Technology and Research), Singapore, 138648 Singapore; 2 Bioinformatics Institute, A*STAR (Agency for Science, Technology and Research), Singapore, 138671 Singapore; 3 Cancer Therapeutics and Stratified Oncology, Genome Institute of Singapore, A*STAR (Agency for Science, Technology and Research), Singapore, 138672 Singapore; 4 National University of Singapore, YLL School of Medicine, Singapore, 119074 Singapore; 5 Present Address: Institute of Pathology, University Hospital Cologne, Kerpener Str. 62, 50937 Köln, Germany; 6 Present Address: Division of Biological & Life Sciences, School of Arts and Sciences, Ahmedabad, India; 7 Present Address: Department of Dermatology, University of Minnesota, 516 Delaware Street S.E., Mail Code 98 Phillips-Wangensteen Bldg., Suite 4-240, Minneapolis, Minnesota 55455 USA

**Keywords:** DNA, Gene expression

## Abstract

Current opinion views androgens as the pathogenic driver in the miniaturization of hair follicles of androgenetic alopecia by interfering with the dermal papilla. This cannot be the sole cause and therefore it is important for therapeutic and diagnostic purposes to identify additional pathways. Comparative full transcriptome profile analysis of the hair bulb region of normal and miniaturized hair follicles from vertex and occipital region in males with and without androgenetic alopecia revealed that next to the androgen receptor as well the retinoid receptor and particularly the PPAR pathway is involved in progressive hair miniaturization. We demonstrate the concurrent up-regulation of *PPARGC1a* in the epithelial compartment and androgen receptor in the dermal papilla of miniaturized hair. Dynamic *Ppargc1a* expression in the mouse hair cycle suggests a possible role in regulating hair growth and differentiation. This is supported by reduced proliferation of human dermal papilla and predominantly epithelial keratinocytes after incubation with AICAR, the agonist for AMPK signaling which activates *PPARGC1a* and serves as co-activator of PPARγ. In addition, miRNA profiling shows enrichment of miRNA-targeted genes in retinoid receptors and *PPARGC1α*/*PPARγ* signaling, and antigen presentation pathways.

## Introduction

Male patterned hair loss, or androgenetic alopecia (AGA) is the most common form of hair loss, prominent in males and characterized by progressive miniaturization of hair follicles^1^. Androgen signaling was considered as prominent cause for AGA as castrated men do not exhibit baldness. Dihydrotestosterone (DHT) acts on androgen receptor (*AR*) at the DP and directly induces the expression of growth inhibiting factors such as *TGF-β2*, *DKK1* and *IL-6*^2–4^. Drugs blocking testosterone metabolism including Finasteride have been proven effective against male AGA in more than half of patients^5–7^. However, there is increasing evidence that additional causes may be involved in AGA pathogenesis, particularly in women^8,9^.

Recent studies have shown the involvement of oxidative stress and mitochondrial activity in AGA. The dermal papilla cells display reduced ability in alleviating oxidative stress despite increased levels of catalase and total glutathione^10,11^. Moreover, regulators of energy metabolism and inflammation such as the nuclear Peroxisome proliferator-activated receptors (*PPARγ*, *α*, *β*) are also involved in hair loss^12^. *PPARγ* is mainly expressed in the epidermis and sebaceous glands in the skin^13–15^. In an anagen human hair follicle *PPARγ* expression is detected in the mesenchymal DP cells, epithelial cells of the outer root sheath (ORS), inner root sheath and matrix^16^. It was shown to inhibit keratinocyte proliferation and promotes terminal differentiation^17^. Total and conditional knock out of *PPARγ* in hair follicle bulge stem cells showed similar result where scarring alopecia was observed in mice with the loss of pilosebaceous units and inflammatory cells infiltration^18,19^. Recent studies showed that treatment of hair follicles by agonistic modulators induced entry into catagen with an anti-inflammatory mechanism^20^.

*PPARGC1α* (*PGC1a*) is a transcriptional coactivator which interacts with *PPARγ* in regulating genes in the energy metabolism pathway induced during exercise^21–23^. However, its role in the skin and hair development is not well characterized.

In this study, we provide further in-depth analysis to findings from transcriptome profiling of hair bulbs from AGA patients identified previously^24^. We show the up-regulation of *PGC1a* in the inner and outer root sheath (IRS/ORS) of hair follicles from AGA patients and the dynamic expression in the mouse hair cycle. The expression of *PGC1a* is concomitant with the *AR* up-regulation in miniaturized hair and indicates its involvement in hair development and pathogenesis of AGA. Characterization of miRNA expression profile provided support for *AR*, PPAR, retinoic acid signaling and inflammation in miniaturized hair.

## Results

### Progressive changes in the transcriptome are evident in hair miniaturization

**Figure 1:**
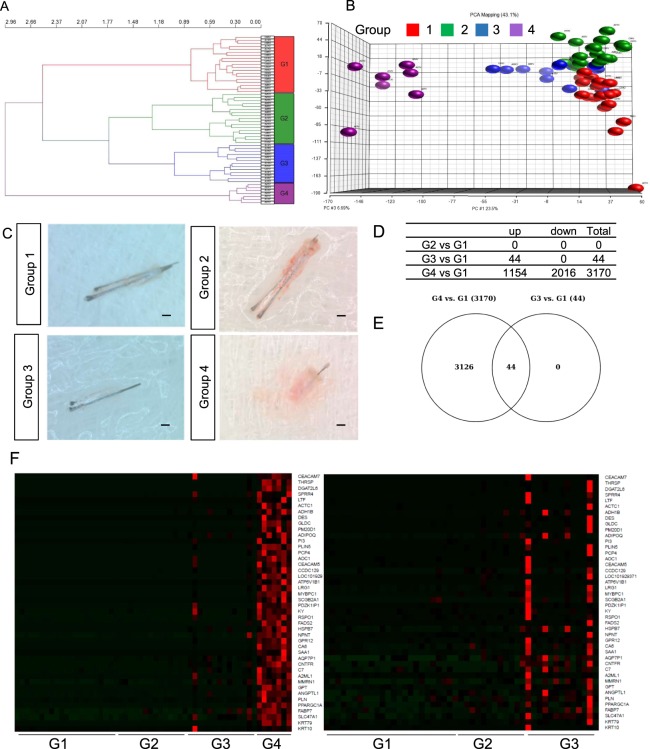
Sample clustering by transcriptome profiling implicates transition in AGA severity. (**A**) Principal component analysis (PCA) plot of mRNA transcriptome profile of FUE samples classified by unsupervised hierarchical clustering. (**B**) Hierarchical clustering of samples according to transcriptome profile. (**C**) Representative images of hair follicles from group 1–4. Scale: 1 mm. (**D**) List of differentially expressed genes between G1 vs G2, G1 vs G3 and G1 vs G4. (**E**) Venn Diagram of differentially expressed genes overlapping between G1 vs G2, G3 and G4. (**F**) Heat map representing expression of genes differentially expressed in G1 vs G3 across G1 to G3 and G1 to G4 samples. Gene expression level are represented by intensity of red color in pixels across different groups of samples.

**Figure 2:**
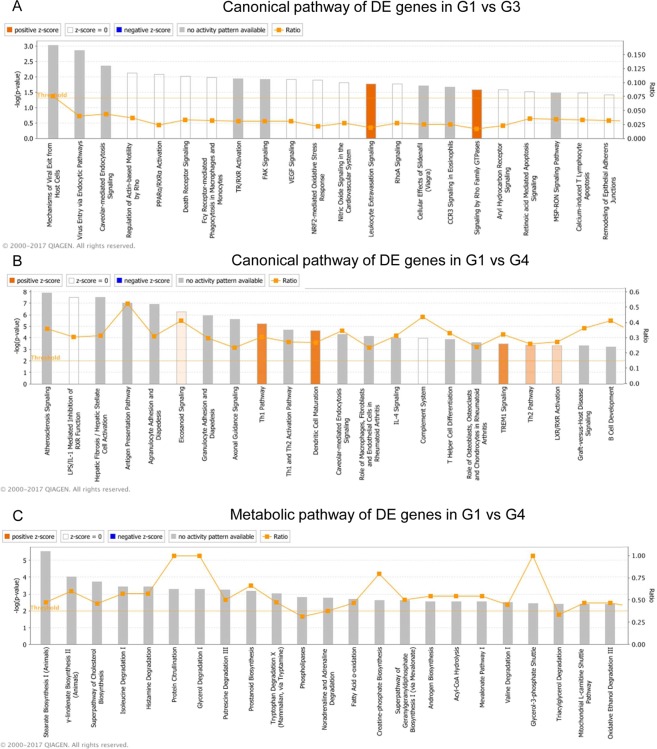
IPA analysis summary of genes were differentially expressed between groups. (**A**) Canonical pathways of genes differentially expressed between G1 and G3. (**B**) Canonical pathways of genes differentially expressed between G1 and G4. (**C**) Metabolic pathways of genes differentially expressed between G1 and G4. The most statistically significant canonical pathways were listed according to −log(p-value) of significance. Orange line represents ratio of genes in enriched pathway against number of genes in the input dataset.

Follicular units from occipital and vertex scalp were extracted in patients and healthy volunteers as previously described^24^ and full transcriptome sequencing of mRNA and miRNA was performed from the hair root region. Clinical classification of AGA according to Hamilton-Norwood scale, location of area sampled and hair follicle morphology (Supp. Fig. 1A–D) resulted in loosely clustered groups. However transcriptome based unsupervised hierarchical clustering (Fig. 1A) resulted in four distinct groups (Fig. 1B). *Group 1* (red in Fig. 1A) comprised mostly occipital and vertex samples from the normal healthy volunteers (CO, CV), (control group). *Group 2* (green) contained patients’ occipital (PO) region, *group 3* (blue) involved patients’ occipital and patients’ vertex (PV) area and they resembled normal hair, but were predominantly shorter. *Group 4* (purple) comprised of follicular units taken from PV exclusively, and all of these are miniaturized hair follicles (Fig. 1C). In the differential gene expression analysis between group 1 and group 2, 3 and 4; there were no differentially expressed genes between group 1 and 2, while we identified 44 and 3170 genes differentially expressed in Group 1 vs Group 3 and Group 1 vs Group 4 respectively (Fig. 1D). It is interesting that follicles under group 3 show transcriptomic changes compared to Group 1 follicles prior to major morphological changes. All up-regulated genes in the Group 3 vs Group 1 comparison were expressed in group 4 samples at much higher levels than those in group 3 (compared to group 1); indicating a transition in group 3 from a healthy hair follicle (group 1 and 2) to a affected, miniaturized hair follicle in group 4 (Fig. 1E,F). Ingenuity Pathway Analysis (IPA) analysis implicated involvement of *PPAR/RXRα* pathway, death receptors and mechanisms for viral-host response in group 3 (Fig. 2A). A myriad of pathways were enriched in group 4 follicles including eicosanoid signaling, antigen presentation and LXR/RXR activation and particularly PPAR signaling (Fig. 2B). PPAR is controlling fatty acid metabolism and its up-regulation in hair bulb was closely related to hair miniaturization and concurrent *AR* up-regulation. Furthermore, IPA analysis of metabolic processes identified the up-regulation of processes including fatty acid oxidation, stearate biosynthesis and prostanoid synthesis (Fig. 2C).

### *PGC1a* expression is elevated in the IRS and ORS of patient vertex hair

**Figure 3:**
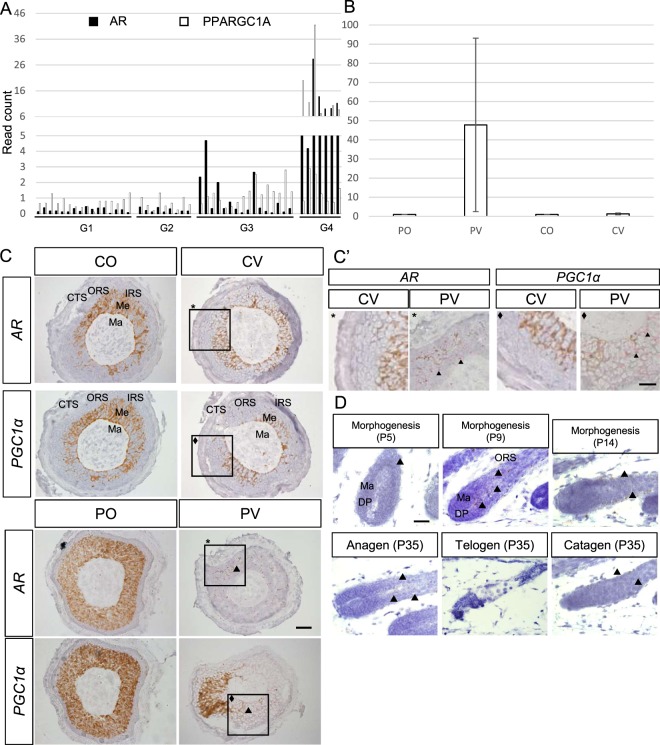
Identification of *PGC1α* as candidate gene involved in AGA. (**A**) *AR* and *PGC1α* transcript expression in FUE samples, black bar represents *AR* transcript read count, white bar represents *PGC1α* transcript read count. (**B**) Validation of *PGC1α* expression by RT-qPCR, fold change between values are normalized to CO samples (PO: 1.08, PV: 47.77, CV: 1.27), p = 0.22 (PV vs CO), results are depicted as mean ± SE, n = 3 per group. (**C**) *In situ* hybridization of *AR* and *PGC1a* in PV, PO, CV and CO samples. Hair follicle sectioned across matrix cells, scale: 20 µm. Arrowhead indicate *in situ* hybridization signals. Square indicate magnified area. Ma: matrix, Me: melanocyte, IRS: inner root sheath, ORS: outer root sheath, CTS: connective tissue sheath. the brown color represents native melanin present in the hair shaft. (**C’**) Higher magnification of AR and PGC1a staining in PV and CV samples. Scale: 10 µm. (**D**) *In situ* hybridization of *Pgc1α* expression in hair follicles of mice at morphogenesis, anagen, catagen and telogen phase of the hair cycle. Scale: 20 µm. Arrowhead indicate *in situ* staining signals in brown.

We identified *PGC1a*, a master regulator for mitochondrial biogenesis^25^, up-regulated in the hair bulb of progressively miniaturized hair samples. *PGC1a* has been shown to interact with *PPARγ* and the retinoid receptor *RARa* in thermogenesis^26^. Interestingly, the transcript levels of *PGC1a*, *PPARγ* and *RAR-a* were found to be concomitantly up-regulated with *AR* expression (Fig. 3A and Supp. Fig. 2A,B). Validation of *PGC1a* expression in control and AGA patients by RT-qPCR confirmed the up-regulation in patients’ vertex follicular hair root compared to patients’ occipital and control samples but with high inter-individual variation (Fig. 3B). *In situ* hybridization showed *PGC1a* and *AR* expressions (red) in the epithelial IRS and ORS cells of patient vertex (PV) samples were elevated compared to all other samples (CO, CV and PO), where they could hardly be detected (Fig. 3C,C’).

### Dynamic *Pgc1α* expression throughout the hair cycle

To assess the role of mouse *Pgc1α* in normal hair development, we performed *in situ* hybridization for *Pgc1α* mRNA in mice to characterize the pattern of expression in different stages of hair cycle (Fig. 3D). In post-natal day 5 mouse hair follicles at morphogenesis stage 5, *Pgc1α* mRNA expression was detected in the IRS of the hair follicle (Fig. 3D; see arrowhead). At P14, as follicles proceed to full maturation, *Pgc1α* expression shifted to the ORS (Fig. 3D; see arrowhead). In fully matured hair at P35, *Pgc1α* was expressed in the epithelial cells of the IRS in anagen, in the ORS of the catagen hair and was not detected in the telogen hair (arrowhead). This distribution pattern was consistent with the expression of *PGC1a* in our human hair follicles at the ORS. Interestingly, *Pgc1α* expression could not be detected in the mesenchymal, *Versican* positive DP cell population at all stages studied (Supp. Fig. 3).

The role of *Pgc1α* in hair development was studied by assessing hair morphology and progression of hair cycle in global *Pgc1α* KO mice^27^. *Pgc1α* KO mice display some postnatal lethality, but the hair morphology and histology in surviving mice was similar to wildtype animals (data not shown). Subsequent to hair morphogenesis, we did not observe significant differences in hair cycle progression (Supp. Fig. 4) brought by knocking out *Pgc1α* as assessed by hair cycle score^28^.

### miRNAs targeting *AR* and PPAR signaling pathways were differentially expressed in miniaturized hair

miRNAs negatively regulate the expression of their target gene post-transcriptionally and are involved in regulating hair development (reviewed in^29^). To gain further insight into the miRNA signatures in AGA, we evaluated the miRNA expression profile in the hair bulb samples from patients and controls. Hierarchical clustering of samples according to miRNA expression profile revealed 4 distinct arms. Equivalent to the Group 4 population in mRNA clustering, the miniaturized follicles formed a distinct cluster according to miRNAs expression (Supp. Fig. 5).

**Figure 4:**
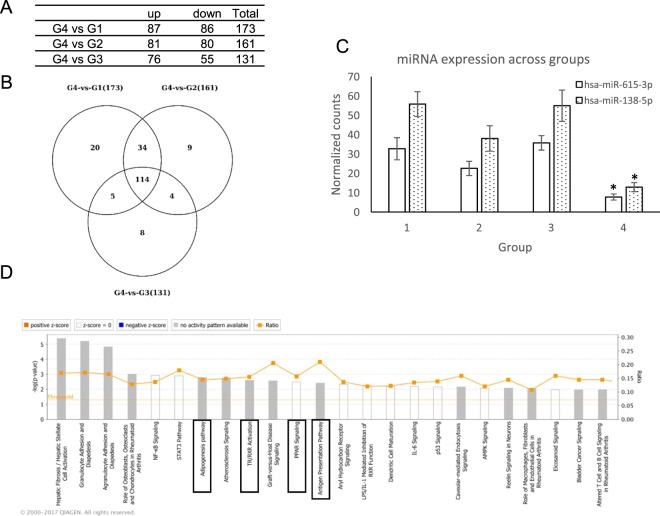
miRNA seq analysis of samples reveal differentially expressed miRNA targeting *AR* and *PGC1α*. (**A**) List of miRNAs differentially expressed in G1 vs G4, G2 vs G4 and G3 vs G4 comparison. (**B**) Venn diagram of overlapping DE miRNA across groups. (**C**) Expression value of miR-138-5p and miR-615-5p across sample groups. Data represented as mean ± SD. *p < 0.05 in G4 compared to all other groups. (**D**) Summary IPA miRNA Target Filter analysis of DE genes from G1 vs G4 comparison superimposed onto experimentally verified and high-confidence targets reported for DE miRNAs. The most statistically significant canonical pathways were listed according to −log(p-value) of significance. Orange line represents ratio of genes in enriched pathway against number of genes in the input dataset.

To investigate how miRNA is associated with the corresponding differentially expressed target mRNA in hair miniaturization, we next analyzed miRNA expression in samples grouped on the basis of mRNA expression profile. No miRNA differential expression was detected in the comparison between G2 vs G1 and G3 vs G1. While G4 vs G1, G4 vs G2 and G4 vs G3 yielded 173, 161 and 131 differentially expressed miRNA respectively, where 114 miRNAs were commonly differentially expressed in all comparisons (Fig. 4A,B). To investigate the association between hair miniaturization and putative miRNA target genes, IPA comparative analysis integrating differentially expressed miRNAs and genes in the Group 4 vs Group 1 was performed. The miRNA target analysis reports the target genes for a miRNA from several sources. Those DE genes that were reported as targets by IPA and showed as expected an opposite trend of expression compared to the DE miRNA, considered as the “actual miRNA targets”. The analysis revealed involvement of target genes were enriched in adipogenesis pathway, PPAR signaling, TR/RXR activation and antigen presentation pathways (Fig. 4C). Thereby miRNA expression analysis provided consistent data and support for the differential mRNA expression in G4 samples. We were particularly interested in the “Androgen Signaling” and FXR/RXR activation pathways and found several miRNAs targeting genes involved in these two pathways (Table 1). We identified two miRNAs, his-miR138-5p and hs-miR615-3p which were preferentially bound on *PGC1α* and *AR* transcripts respectively were down-regulated in G4 samples (Fig. 4D). This supports a possible mechanism of miRNA regulating DE mRNAs in AGA. We also find high expression of miR128-3p, miR500a-3p and let-7a-5p which were differentially expressed in dermal papilla cells upon DHT treatment^30^.

**Table 1:** DE miRNAs in Group 4 vs Group 1 comparison predicted to target genes in the AR and PPAR signaling pathways.

MiRNA ID	Expr p-value	MIRNA Expr Fold Change	Symbol	MRNA Expr log Fold Change	Pathway
hsa-miR-98-5p	0.00000126	−2.271	PPARGC1B	2.893	LPS/IL-1 Mediated Inhibition of RXR Function
hsa-miR-301b-3p	0.0000118	−6.39	PPARG	6.658	Adipogenesis pathway, ERK/MAPK Signaling, FXR/RXR Activation,
hsa-miR-138-5p	0.00781	−5.901	PPARGC1A	8.296	AMPK Signaling, Estrogen Receptor Signaling, FXR/RXR Activation
hsa-miR-27b-3p	6.84E-07	−2.189	PPARG	6.658	Adipogenesis pathway, ERK/MAPK Signaling, FXR/RXR Activation,
hsa-miR-92a-1-5p	0.00329	−5.08	PPARGC1A	8.296	AMPK Signaling, Estrogen Receptor Signaling, FXR/RXR Activation
hsa-miR-92a-1-5p	0.00329	−5.08	PPARGC1B	2.893	LPS/IL-1 Mediated Inhibition of RXR Function
hsa-let-7d-3p	0.00313	−2.569	PRKACB	2.166	AMPK Signaling, Amyloid Processing, Androgen Signaling
hsa-miR-128-3p	0.00111	−2.576	PRKD1	2.769	14-3-3-mediated Signaling, Aldosterone Signaling in Epithelial Cells, Androgen Signaling
hsa-miR-138-5p	0.00781	−5.901	GNG2	2.87	Androgen Signaling, Antiproliferative Role of Somatostatin Receptor 2
hsa-miR-148b-3p	0.00000885	−2.159	MRAS	2.83	14-3-3-mediated Signaling, Actin Cytoskeleton Signaling
hsa-miR-182-5p	0.0000627	−2.718	PRKACB	2.166	AMPK Signaling, Amyloid Processing, Androgen Signaling, Axonal Guidance Signaling
hsa-miR-3656	0.00000101	5.481	POLR2L	−1.226	Androgen Signaling, Assembly of RNA Polymerase II Complex, CREB Signaling in Neurons
hsa-miR-539-3p	0.00902	2.178	GNA14	−2.398	Androgen Signaling, Axonal Guidance Signaling
hsa-miR-487b-3p	0.00000964	2.389	SRY	−1.544	Androgen Signaling, Neuroprotective Role of THOP1 in Alzheimer’s Disease
hsa-miR-615-3p	0.00922	−5.078	AR	5.175	Androgen Signaling
hsa-miR-7977	0.00418	−2.261	GNG2	2.87	Androgen Signaling, Antiproliferative Role of Somatostatin Receptor 2, Axonal Guidance Signaling

### Impaired keratinocyte and DP cell proliferation upon AICAR treatment

**Figure 5:**
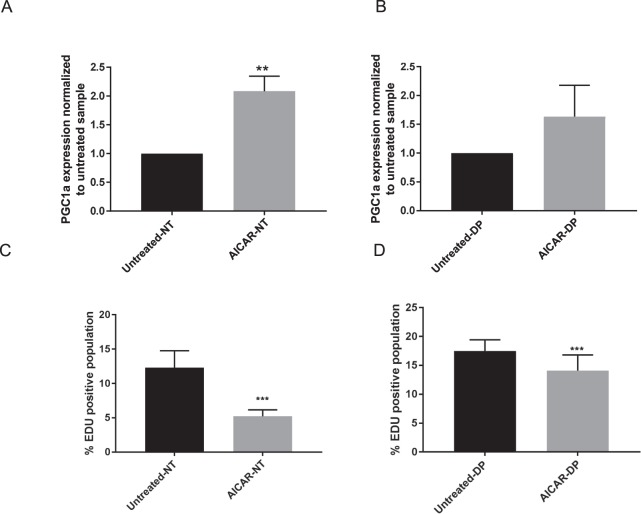
AICAR treatment of immortalized keratinocytes NTERTs (NT) and dermal papilla (DP) *in vitro* for 24 hours. *PGC1α* expression level in (**A**) NT, (p = 0.005) and (**B**) DP cells (p = 0.06) by qPCR compared to untreated control. EdU positive cells by flow cytometry after EdU labelling for 2 hours of AICAR treated (**C**) NT and (**D**) DP cells compared to untreated control. Data represented as mean ± SD. n = 3 per group. p-value for difference between untreated and treatment group: NTERTs p < 0.0001, DP p = 0.043.

To investigate the mechanism of *PGC1α* in causing hair miniaturization, we then analyzed the impact of elevated *PGC1α* expression on immortalized epithelial keratinocytes and mesenchymal DP cell proliferation. To stimulate *PGC1α* expression *in vitro*, cells were treated with AICAR (1 mM), an AMPK stimulator well known to induce *PGC1α* in dermal fibroblasts and keratinocytes^31,32^. *PGC1α* expression was induced by 2 fold and 1.6 fold immortalized keratinocyte and DP cells respectively after AICAR treatment for 24 hours (Fig. 5A). AICAR-stimulation resulted in a significant decrease of cell proliferation from 11.36% (Untreated) to 5.185% (AICAR treated) in NTERTs and from 17.43% (Untreated) to 14.09% (AICAR treated) in DP cells as measured by DNA-incorporation of EdU label (Fig. 5B).

## Discussion

The progressive nature of hair miniaturization in AGA has been demonstrated with the discovery of intermediate hair follicles which presents altered morphology^33^. Following up on the progressive changes in the transcriptome profile is identified previously^34^; we find a discrepancy between transcriptome profile in each hair follicles and hair morphology. Classification by subjective assessments using AGA severity, regional differences and hair morphology did not yield distinct clusters of healthy and diseased hair follicles based on the transcriptome profile. This also implicates the hair bulb transcriptome was not dependent on degree of balding and embryonic origins between the vertex and occipital regions. The classification based on unsupervised hierarchical clustering of transcriptomic profile provided a more objective assessment of hair follicle condition in comparison to previous methods. The changes in transcriptome precede actual miniaturization and presents an opportunity for early diagnostic and treatment window.

Our study shows dynamic P*GC1a* expression in hair cycle and elevation in the progression of hair miniaturization in AGA. *PGC1a* function has been well characterized in high energy consumption tissues including the muscle, heart and liver where it acts as the master inducer of mitochondrial biogenesis and elevates oxidative metabolism^35^. Its role in the hair is poorly understood. Our study has provided the first insight into the distribution and role of *PGC1a* in hair growth. This is particularly important to explain the miniaturization and hair loss phenotype in female pattern AGA in which androgen signaling is less involved^36,37^. The dynamic expression of *Pgc1a* in the differentiating zone of mouse hair during anagen suggests it may be involved in orchestrating energy metabolism and the very complex process of hair differentiation. Indeed mitochondrial activity is found to peak during hair growth where mitochondria are elongated and oxidative phosphorylation predominates over glycolysis in the proliferating and differentiating cells^38^. However, a conclusive answer has yet to be established for its role in hair growth. Compensation by other genes in the PPAR family in regulating hair growth and changes in other tissues might account for the lack of phenotype in *Pgc1a* KO mice. Skin-specific manipulation of *Pgc1a* expression would be important to further decipher the role of *Pgc1a* in hair growth. Similar to previous studies on keratinocytes^32^ we have observed that treatment with AICAR induce *PGC1a* and diminished proliferation in epithelial cells (N/TERT keratinocytes) and much less in dermal papilla cells, which supports our hypothesis that *PGC1a* may be a mediator for hair follicle miniaturization in AGA in the epithelial compartment of the hair follicle. AICAR is known to induce *PGC1a* expression and activity through phosphorylation. AICAR also induces the activity of AMPK signaling and other gene in the energy metabolic pathway, therefore further studies have to be performed to separate the role of *AR* and *PGC1a* in hair differentiation and miniaturization.

Studies on *PPARγ* suggested that it inhibits hair growth, possibly through the anti-inflammatory and promotion of mitochondrial activity^20,39^. On the contrary, *NRF2*, a downstream target of *PGC1a* is suggested to alleviate oxidative stress-induced catagen and growth inhibition^40^. Therefore it is likely the PPAR signaling can modulate hair growth in multiple pathways and can be context dependent.

The fact that *PGC1a*, as a master regulator for mitochondrial biogenesis and ability to bind to large array of genes such as *RXRα*, estrogen receptors and androgen receptor adds to the complexity of deciphering its role in hair growth^41–43^. Further work needs to be done to distinguish if *PGC1a* is involved in AGA pathogenesis or plays a protective role against AGA. The interplay between the *PGC1a*/*PPARγ* and retinoid pathways in our analysis is of particular interest, because treatment with retinoids leads to a well-known temporary hair loss as side effect^44^ and shows the importance of these interacting networks in hair homeostasis and its clinical relevance. Low level of *AR* and *PGC1a* expression detected in the patient IRS and ORS suggested the possible interaction between them in the hair, an interaction which has been described in the context of prostate cancer. Androgens-mediated AMPK signaling elevates *PGC1a* expression and activity which promotes energy metabolism through glycolysis and the OXPHOS pathway, resulting in enhanced tumor cell growth^45^.

Changes of miRNA expression in DP cells isolated from balding scalp and cells treated with DHT has been reported previously^30,46^. miR-221, miR-125b, miR-106a, miR-410 are up-regulated in DP cells of balding scalp, are known to target genes involved in prostate cancer development. It is possible these candidate miRNAs are implicated in androgen-mediated hair loss. In DHT treated DP cells, miRNA targeting genes involved in oxidative stress and apoptosis are differentially expressed. This finding supports a potential miRNA-regulated mechanism in the occurrence of oxidative stress-associated senescence in DP cells isolated from balding patients^10^. In this study we revealed that miRNAs targeting genes involved in PPAR signaling and antigen presentation are differentially expressed in miniaturized hair samples. This observation is consistent with the mRNA expression analysis. Interestingly, the miRNA analysis further pointed to the involvement of immune response in AGA. Micro-inflammation has been well described in AGA with the occurrence of pro-inflammatory cytokines, T-cell and macrophage infiltration and perifollicular sheath fibrosis evident in the progress (reviewed in^47^). Their relation with hair miniaturization has to be further delineated.

In conclusion, our findings provide a new link between *PGC1a* and hair development. Further study will be required to understand the mechanisms regulating hair growth and AGA pathogenesis albeit the complexity in PPAR signaling pathways. It is important to establish the role of interactions between *AR*, PPAR and retinoid pathways in hair differentiation. The new insights and knowledge of this publication can lead to new ways for AGA prediction, early diagnosis and new treatments to delay or avoid hair miniaturization. In any case this publication proves that the hair growth and differentiation is a very complicated and multifaceted process and it requires knowledge of all the key players to understand and support hair growth.

## Materials and Methods

### mRNA and miRNA extraction for sequencing analysis

Thirty men were recruited with informed consent for this study under the ethics approval and consent to participate – Singapore National Healthcare Group - Domain Specific Review Board (NHG-DSRB) number: “2012/00488 Transcriptome and genome analysis of human scalp biopsies of androgenetic alopecia before and after topical laser treatment”. 20 AGA patients and 10 healthy volunteers were included. RNA was extracted from the lower portion of the hair follicle using Allprep micro kit (Qiagen) as previously described^24^. miRNA has been isolated with additional wash with 100% ethanol from flow through of RNA extraction.

### Library construction and sequencing analysis

Library construction was performed with TruSeq Small RNA Library Prep kit (Illumina). RNA sequencing was performed on HiSeq2000 platforms, the sequencing, mapping and quantification of reads are described in supplementary materials and methods. The clustering of the samples was checked using the TMM normalised expression values. mRNA seq samples were grouped into four groups based on their transcriptome. These four groups were compared using the glmTreat function in edgeR as follows:

$${\rm{G}}4\,{\rm{vs}}.\,{\rm{G}}1,\,{\rm{G}}4\,{\rm{vs}}.\,{\rm{G}}2,\,{\rm{G}}4\,{\rm{vs}}.\,{\rm{G}}3,\,{\rm{G}}3\,{\rm{vs}}.\,{\rm{G}}1,\,{\rm{G}}3\,{\rm{vs}}.\,{\rm{G}}2,\,{\rm{G}}2\,{\rm{vs}}.\,{\rm{G}}1$$

A log-fold change (logFC) cut-off of 1 and p-value (with FDR) <0.05 cut-off was used to obtain the differentially expressed genes. Visualization of transcriptome profile clustering and PCA plot is performed with Partek® Genomics Suite.

Grouping of samples based on morphology into healthy, intermediate and miniaturized samples as assessed by three independent physicians. Grouping of samples by measurements was based on measuring the length of extracted hair follicles from skin surface to the hair bulb. Hair follicular units longer than 4 cm were considered healthy, 3–4 cm were considered intermediate and >3 cm were considered miniaturized.

### Identification of enriched canonical pathways

The differentially expressed genes obtained from G4 vs. G1 and G3 vs. G1 comparisons were uploaded into Ingenuity Pathway Analysis (IPA) software and a core analysis was carried out for each of these two lists. A threshold score of p-value 0.05 corresponding to a significance score of 1.3 was used for G3 vs. G1 DE list and a threshold score of p-value 0.01 corresponding to a significance score of 2 was used for G4 vs. G1 DE list.

### miRNA sequencing analysis

Small RNAseq data was obtained from 42 samples using Illumina Truseq smallRNA protocol. Sequencing, mapping and quantification of miRNA sequences are described in supplementary materials and methods. The miRDeep2 normalised expression data for all the miRNAseq samples were combined into one file and analysed further in Partek. The miRNAseq samples were grouped into the four groups (G1–G4) according to the mRNAseq grouping strategy.

The ANOVA model and the following comparisons were carried out to obtain the differentially expressed miRNAs:

$${\rm{G}}4\,{\rm{vs}}.\,{\rm{G}}1,\,{\rm{G}}4\,{\rm{vs}}.\,{\rm{G}}2,\,{\rm{G}}4\,{\rm{vs}}.\,{\rm{G}}3,\,{\rm{G}}3\,{\rm{vs}}.\,{\rm{G}}1,\,{\rm{G}}3\,{\rm{vs}}.\,{\rm{G}}2,\,{\rm{G}}2\,{\rm{vs}}.\,{\rm{G}}1.$$

A two fold change cut-off and a p-value (with FDR) <0.05 cut-off was used.

### miRNA targets analysis and integration of DE miRNA and mRNA

The DE miRNAs list from the G4 vs. G1 comparison were uploaded in IPA and a miRNA Target Filter Analysis was carried out. DE genes list from the G4 vs. G1 comparison was superimposed onto the full set of experimentally verified and high-confidence targets reported for the DE miRNAs. The DE genes that were reported by IPA miRNA Target Filter analysis and that showed an opposite trend of expression as compared to the DE miRNAs were selected for further IPA core analysis. A threshold score of p-value 0.05 corresponding to a significance score of 1.3 was used to obtain significantly enriched canonical pathways for this miRNA target genes set.

### qRT-PCR analysis

qRT-PCR is performed as previously described^34^. Fold change of *PGC1α* expression was calculated by normalizing its Ct value against the *RPL13A* gene using the 2^ΔΔCT^ method. Quantitect Primer assay #24990 (Qiagen) was used to detect human *PGC1α*, *RPL13A* gene was detected with the forward primer: CTCAAGGTCGTGCGTCTGAA and reverse primer: TGGCTGTCACTGCCTGGTACT. Statistical significance was calculated using Wilcoxon sign rank test, p-value smaller than 0.05 was considered significant.

### *In situ* hybridization

Mouse back skin and human FUE samples were fixed in formalin, embedded in paraffin and sectioned at 5 µm thickness. *In situ* hybridization was performed on transverse paraffin sections of the human follicle using ACD RNAscope HD 2.5 duplex assay or 2.5 HD Brown assay with probe HS:VCAN, Hs:AR and HS:PPARGC1a. *In situ* hybridization on mouse skin was performed with Ms:PPARGC1a and Ms:VCAN probes. Staining was visualized in a traditional light microscope (Zeiss Axio Imager Z1 upright).

### Hair cycle study

Mouse skin sections were obtained from Wildtype and P*gc1a* KO mutants obtained from Christoph Handschin (Basel, Switzerland) at designated time points of the hair cycle to assess progression of hair growth. Hair follicles at distinct hair cycle stages were classified and assigned according to hair cycle score system as described^28^. Animals were housed in a conventional facility with a 12 h night/day cycle, with free access to food/water. Experiments were performed on adult male mice in accordance to Swiss federal guidelines for animal experimentation and were approved by the Kantonales Veterinäramt of Kanton Basel-Stadt.

### Cell culture and treatment

Immortalized human keratinocytes (NTERTs) are maintained in KSFM media (Gibco) supplemented with 0.4 mM calcium chloride^48^. Immortalized dermal papilla cells^49^ are maintained in Dulbecco’s modified Eagle’s medium (DMEM, Gibco) supplemented with 10% fetal bovine serum (FBS) and 1% penicillin-streptomycin (10,000 U/ml penicillin and 10 mg/ml streptomycin, PAN-Biotech GmbH, Germany). Immortalized dermal papilla cells are maintained in 10% CO_2_ at 37 °C. 5-Aminoimidazole-4-carboxamide 1-β-D-ribofuranoside (AICAR, Sigma) is administered for 24 hours at 0.5 mM. Dermal papilla cells are cultured in activated charcoal stripped FBS supplemented DMEM one day prior to treatment.

### EdU labeling proliferation assay

Cells are labeled with 10 µM EdU for two hours and then processed and stained according to manufacturer instructions by Click-iT EdU Flow Cytometry Assay kit (Thermo Fisher). Flow cytometry analysis was carried out with BD FACS Celesta (BD Biosciences) at 647 nm, data was analyzed with FlowJo software (TreeStar, Ashland).

### Statistical analysis

Data were analyzed by student’s t-test with the GraphPad Prism software. Data were represented as mean ± SD, statistical significance were represented as *p < 0.05, **p < 0.01, ***p < 0.001.

### Ethics approval and consent to participate

Ethics approval and consent to participate – Singapore National Healthcare Group - Domain Specific Review Board (NHG-DSRB) number: “2012/00488 Transcriptome and genome analysis of human scalp biopsies of androgenetic alopecia before and after topical laser treatment”. All methods were performed in accordance with the relevant guidelines and regulations.

Animal experiments were performed in accordance with Swiss federal guidelines for animal experimentation and were approved by the Kantonales Veterinäramt of Kanton Basel-Stadt.

## Supplementary information

Supplementary information

## Data Availability

Sequencing data will be deposited in GEO-NCBI with publication of manuscript.
