# Supplementary material for: Progressive expression of PPARGC1α is associated with hair miniaturization in androgenetic alopecia
Source: Sci Rep. 2019 Jun 19;9:8771. doi: 10.1038/s41598-019-43998-7 (PMC6584672; doi:10.1038/s41598-019-43998-7)
Supplement: Supplementary file 1 — Supplementary information [file 41598_2019_43998_MOESM1_ESM.pdf]

Title

Progressive expression of *PPARGC1α* is associated with hair miniaturization in androgenetic alopecia

- **Author**
- Bryan Siu-Yin Ho[1], Candida Vaz[2], Srinivas Ramasamy[1], Elaine Guo Yan Chew[3], Jameelah Sheik Mohamed[1], Huma Jaffar[4], Axel Hillmer[3#a], Vivek Tanavde[2#b], Mei Bigliardi-Qi[1#c], Paul Lorenz Bigliardi[1 #c*]
- **Affiliation**
- 1. Experimental Dermatology Group, Institute of Medical Biology, A*STAR (Agency for Science, Technology and Research), Singapore 138648, Singapore
- 2. Bioinformatics Institute, A*STAR (Agency for Science, Technology and Research), Singapore 138671, Singapore
- 3. Cancer Therapeutics and Stratified Oncology, Genome Institute of Singapore, A*STAR (Agency for Science, Technology and Research), Singapore 138672, Singapore
- 4. National University of Singapore, YLL School of Medicine, Singapore 119074, Singapore
- #a. Institute of Pathology, University Hospital Cologne. Kerpener Str. 62, 50937 Köln (Current)
- #b. Division of Biological & Life Sciences, School of Arts and Sciences, Ahmedabad (Current)
- #c Department of Dermatology. University of Minnesota. 516 Delaware Street S.E., Mail Code 98 Phillips-Wangensteen Bldg., Suite 4-240 Minneapolis, Minnesota 55455 (Current)
-
- ***Corresponding author**
- Paul Lorenz Bigliardi,
-
- #06-11 Immunos, 8A Biomedical Grove, Singapore 138648,
  +65- 6772 2002 ; paul.bigliardi@gmail.com
-
- Current correspondence: Department of Dermatology
- 516 Delaware Street S.E., Mail Code 98, Phillips-Wangensteen Bldg., Suite 4-240, Minneapolis, Minnesota 55455.
- Academic Office: (612) 625-8625, pbigliar@umn.edu

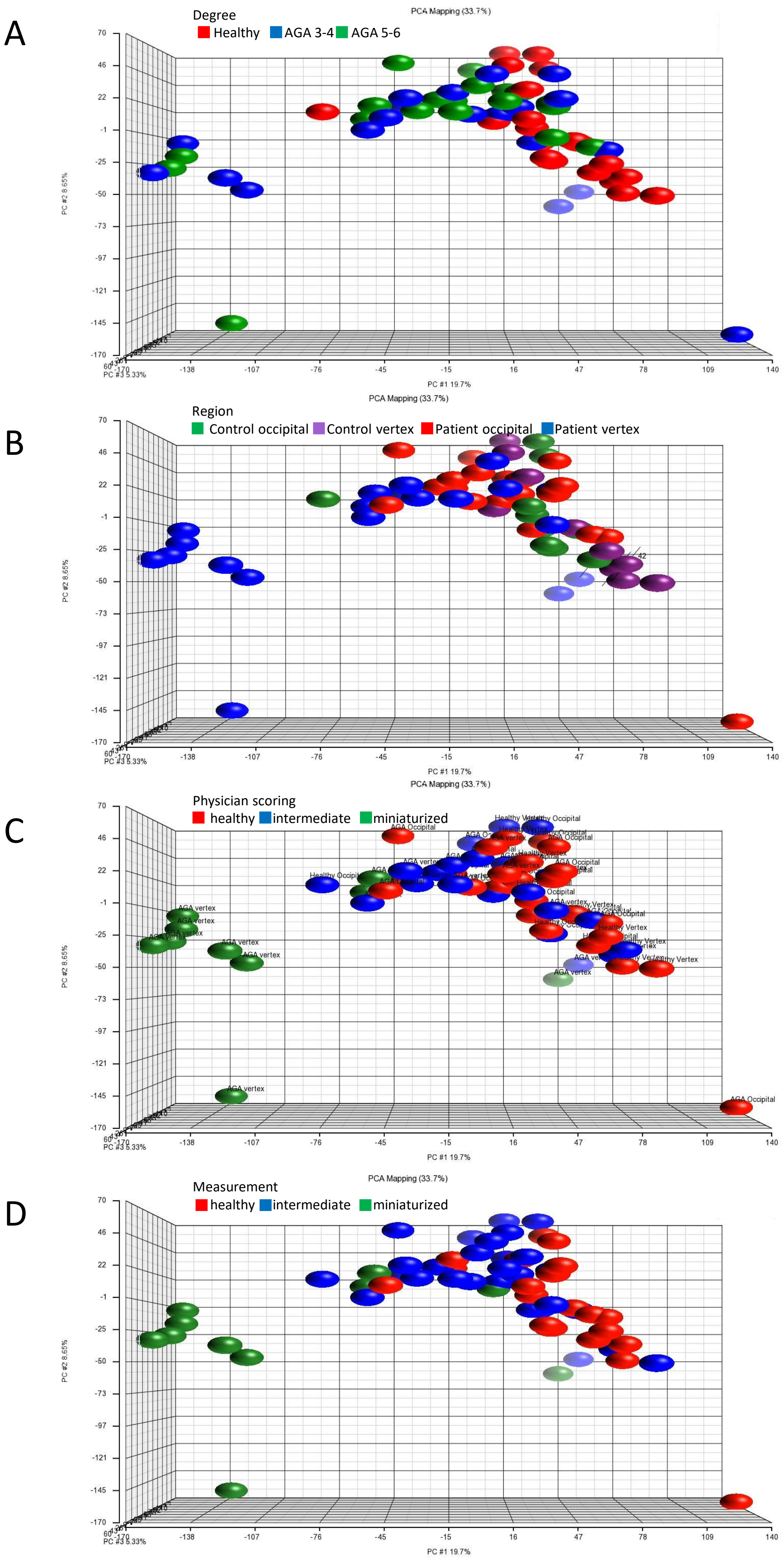

Supp. Figure 1. Sample clustering by transcriptome profiling implicates transition in AGA severity
Principal component analysis (PCA) plot of transcriptome profile of FUE samples classified by A. patient AGA severity in Hamilton Scale by control, below and above Grade four B. Localization of FUEs by vertex and occipital region in patient and control C. physician assessment of FUE morphology D. Length of FUE

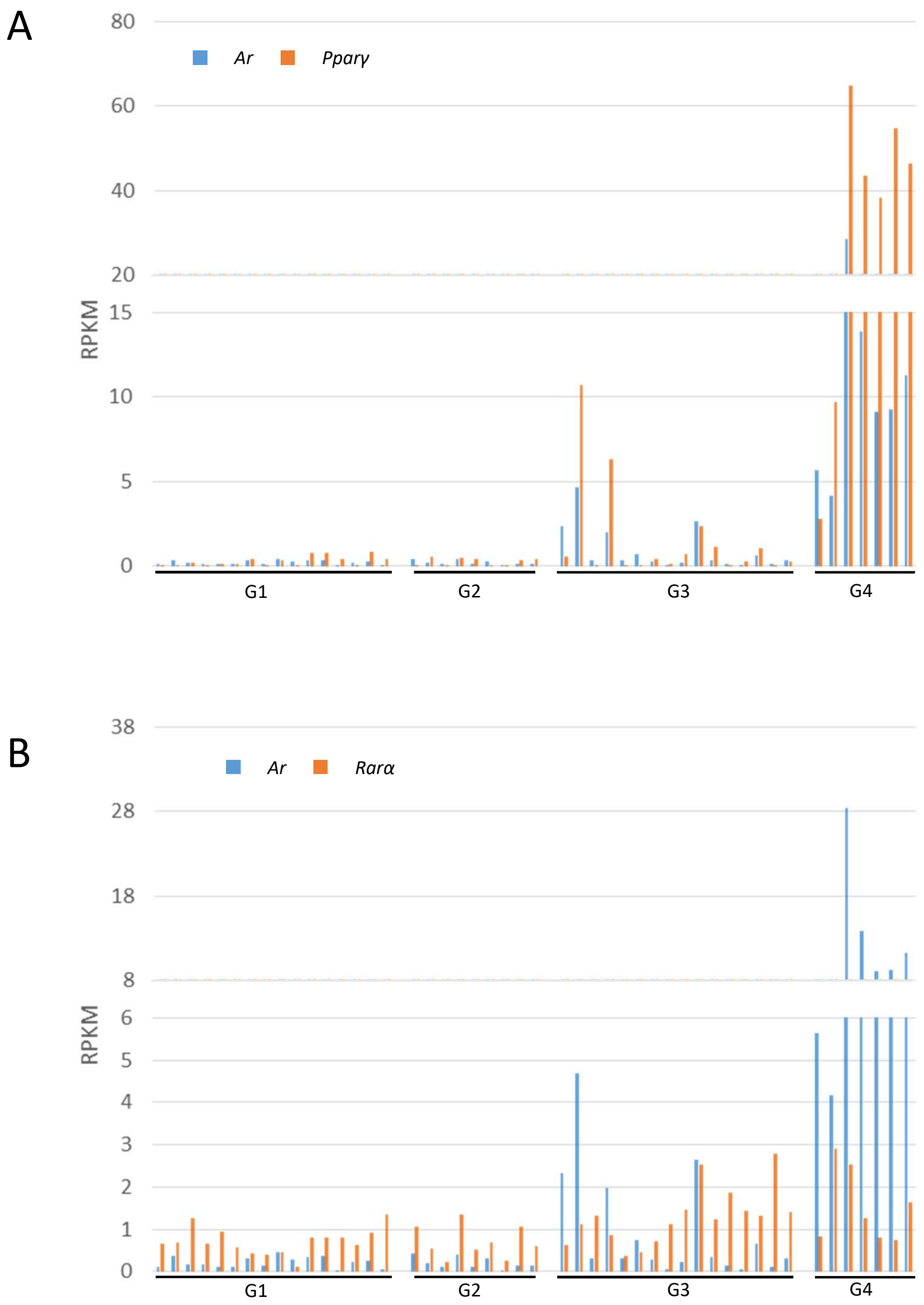

Supp Figure 2. Identification of *Ppar* signaling pathway members as candidate genes involved in AGA A. *AR* and *PPARγ* transcript expression in FUE samples. B. *AR* and *RARA* transcript expression in FUE samples.

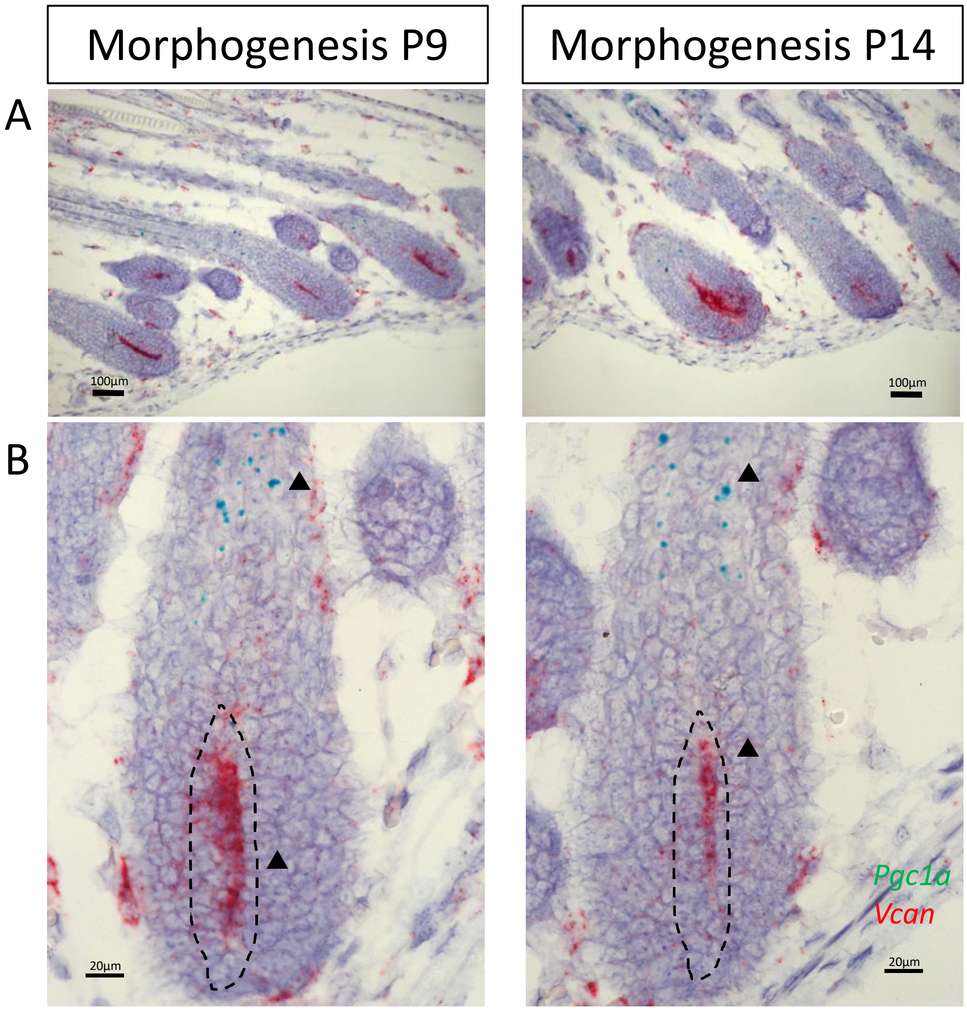

Supp Figure 3 *Pgc1a* and *Vcan* expression in mouse hair follicle during morphogenesis
A. In situ hybridization of *Pgc1a* (green) and *Vcan* (red) indicated by arrow heads on mouse back skin hair follicle on P9 and P14 of hair morphogenesis. Scale: 100µm B. Higher magnification of neighboring hair follicles, dermal papilla region is outlined in dotted lines. Scale: 20µm.

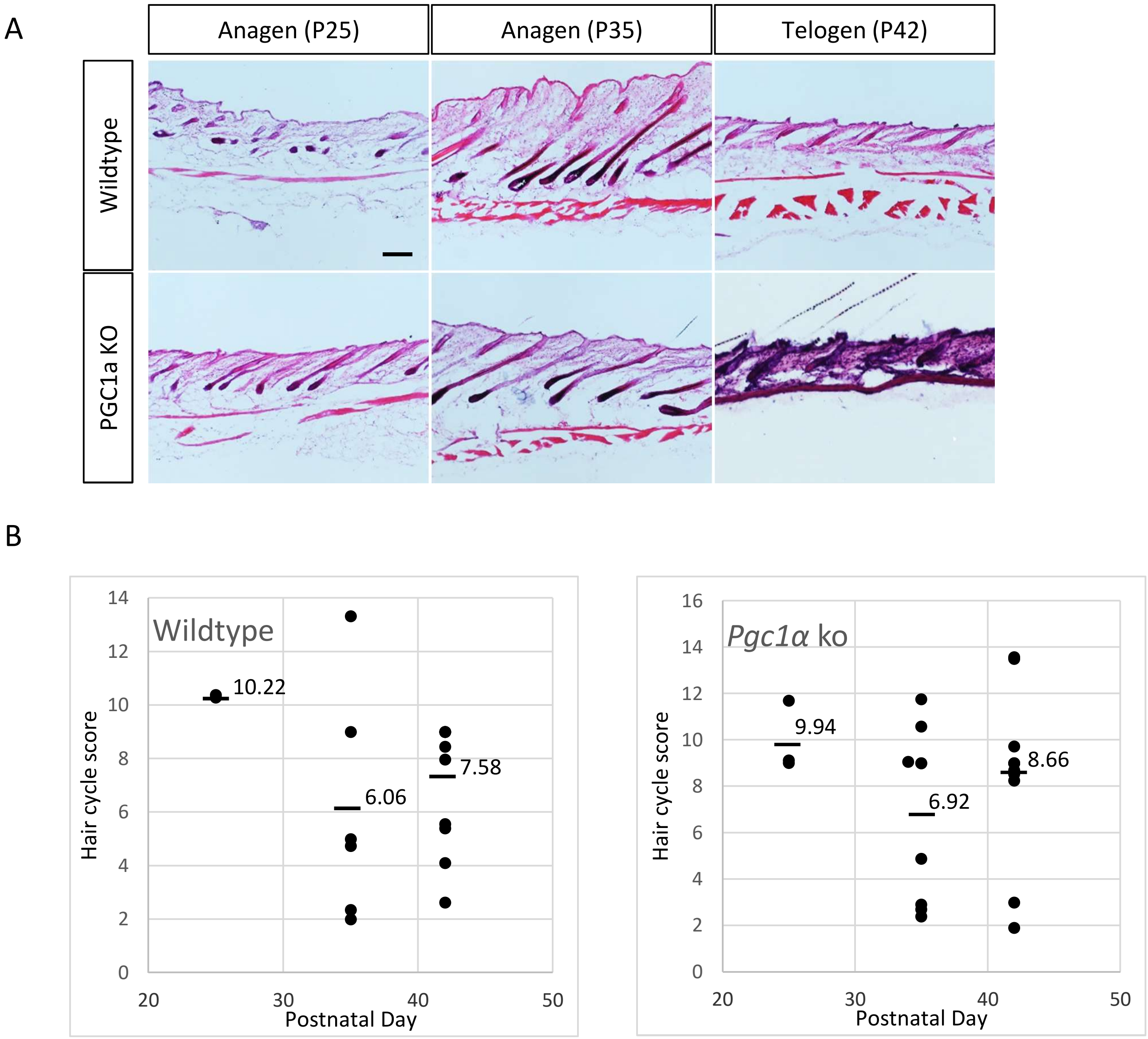

Supp Figure 4. Hair cycle progression in wildtype and *Pgc1α* knockout mice.
A. H&E staining of back skin from wildtype and *Pgc1a* KO mice on P25, P35 and P42, per each time point. Scale: 100µm. B. Hair cycle score in wildtype and *Pgc1a* KO mice assessed as described previously (3). Bar represents mean score per group. 3, 9 and 13 mice were harvested at P25, 35 and 42 respectively, 50 hair follicles were assessed per mouse.

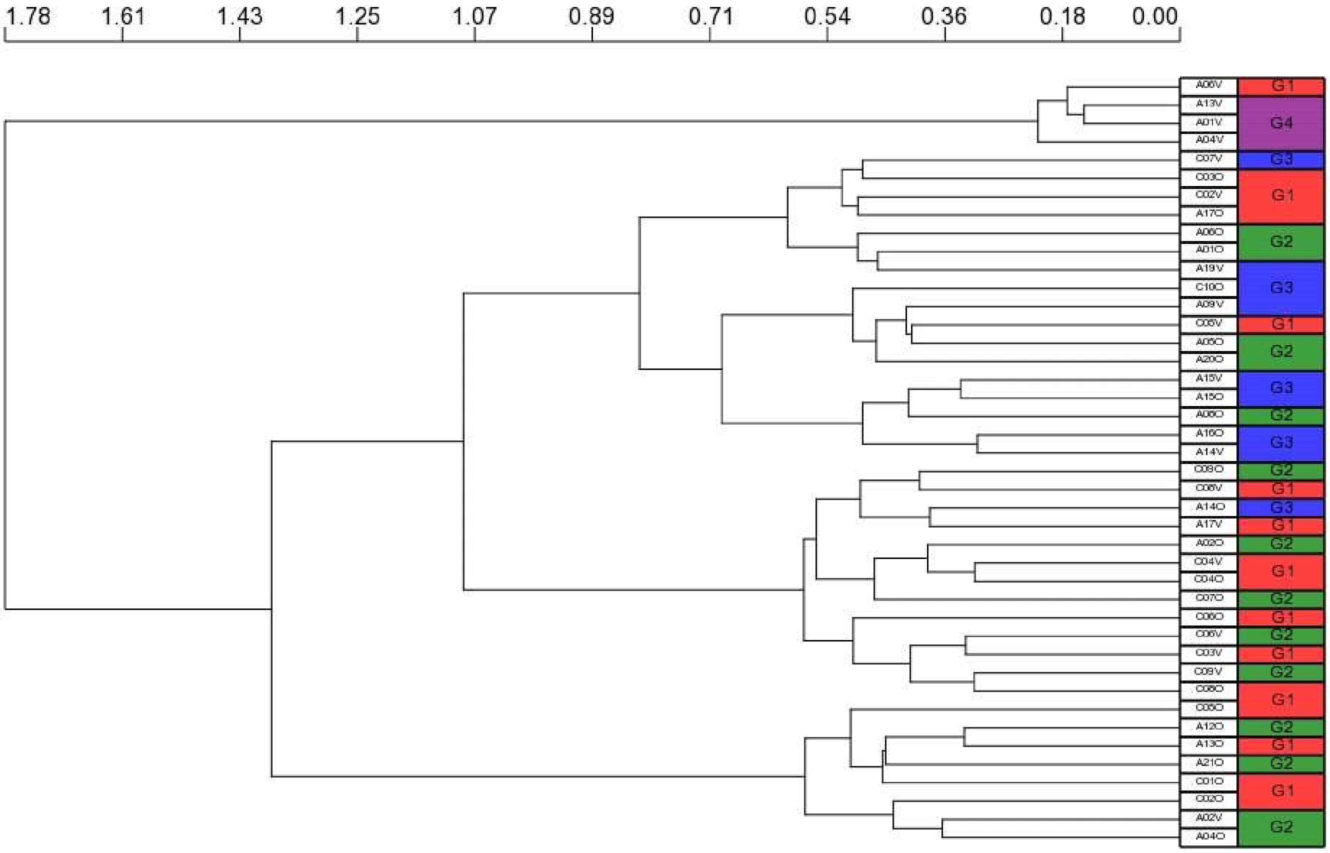

Supp Figure 5 Hierarchical clustering of samples according to miRNA expression profile
Samples were clustered according to miRNA expression profile, samples were labelled according mRNA expression profile grouping.
